# Global Distribution and Comparative Resistance Profiles of *Escherichia coli* Isolated From Bovine Meat and Meat Products: Evidence From Network Meta‐Analysis

**DOI:** 10.1002/fsn3.72136

**Published:** 2026-07-26

**Authors:** Yaser Khorram Del, Peyman Mahmoudi, Amir Rashidi, Skala Idrees Hama Faraj, Jalal Rostamzadeh, Mohammad Razmkabir

**Affiliations:** ^1^ Department of Animal Production, Kifri Technical College Garmian Polytechnic University Kifri Iraq; ^2^ Department of Animal Science, Faculty of Agriculture University of Kurdistan Sanandaj Iran

**Keywords:** antimicrobial resistance, bovine meat, *E. coli*, network meta‐analysis

## Abstract

*Escherichia coli* is a pivotal indicator organism for monitoring antimicrobial resistance (AMR) in the food chain. This study investigates global resistance profiles in *E. coli* isolates recovered from bovine meat and its derivatives employing a network meta‐analysis (NMA). A Bayesian NMA was created by collating 100 studies from 41 countries that included 221,420 *E. coli* isolates. Resistance to 39 different antimicrobials was analyzed using the Surface Under the Cumulative Ranking Curve (SUCRA) and odds ratios (OR). Node‐splitting was used to assess inconsistency between studies, and future trends were estimated using the Prophet time series forecasting method. The results of the NMA indicate that carbapenems and fourth‐generation cephalosporins had the largest SUCRA values of 0.0008 and 0.1170, respectively, showing the least amount of resistance to imipenem and meropenem. Conversely, highest resistance levels were documented for penicillins (SUCRA = 0.974) and diaminopyrimidines (SUCRA = 0.908) classes, with penicillin (0.999), doxycycline (0.951), and amoxicillin (0.942). Pathogenic strains had a significantly higher proportion of resistance (32.35%) compared with commensal strains (24.09%). In addition, developing countries had nearly twice the percentage of resistance (31.73%) as did developed countries (17.03%). Estimates for resistance at the global level indicate that resistance will increase from 32% to 63.44% by 2040. The ability of widely used antimicrobials to maintain their effectiveness compared with a large number of common agricultural antimicrobials that have very high failure rates illustrates the importance of selective pressure and regulatory oversight. Strengthening of international stewardship and surveillance to prevent further spread of resistant foodborne pathogens is critical.

## Graphical abstract

Schematic overview of the study and its principal findings. *E. coli* isolated from bovine meat and meat products was evaluated for antimicrobial resistance using Bayesian network meta‐analysis. The graphical abstract highlights the antimicrobial resistance ranking based on SUCRA, showing high resistance to penicillin, doxycycline, amoxicillin, oxytetracycline, and ampicillin, and low resistance to amikacin, levofloxacin, cefepime, meropenem, and imipenem. It also illustrates the higher resistance observed in pathogenic than non‐pathogenic *E. coli* , in developing than developed countries, and the geographical distribution of resistance across continents.

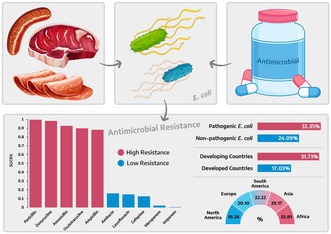

## 1 Introduction

*Escherichia coli* ( *E. coli* ) is a diverse microorganism both morphologically and genetically, being the primary resident of the gastrointestinal tracts of mammals and birds (Yu et al. 2021). Most *E. coli* strains occur as beneficial commensals, forming a substantial part of the healthy gut microflora. Some *E. coli* strains, however, acquired their pathogenic traits, and they are classified into two major categories: diarrheagenic (intestinal) and extraintestinal. These pathogens represent various types of pathotypes and hybrids (Geurtsen et al. 2022). Clinical presentations of these pathogens vary greatly. Extraintestinal opportunistic strains usually occur without causing any harm to the host, and they can become pathogenic only after translocation to some other body areas like the urinary system or the bloodstream, while the obligate intestinal pathogens are specialized to trigger an illness in the digestive system. Depending on their virulence potential, clinical presentations may be as different as asymptomatic infection and deadly consequences (Govindarajan et al. 2024). As stated by the World Health Organization (2024), *E. coli* is still the number one cause of morbidity and mortality around the world, especially affecting vulnerable populations such as children and elderly people. Importance of *E. coli* in foodborne outbreaks along with the rising trend of the organism's resistance to antimicrobials led to its wide implementation as a marker of food safety.

The global average consumption of meat and related products per capita annually stands at 9.39 kg, with the Americas having the highest consumption rates at 28.46 kg and Africa having the lowest at 5.04 kg (Food and Agriculture Organization of the United Nations 2023). Contamination by *E. coli* , especially the strain which produces Shiga toxin known as O157:H7 in meat products, poses serious health risks to the consumer, such as bloody diarrhea, hemolytic uremic syndrome (HUS), and kidney failure (Gambushe et al. 2022). Such contamination is possible during several stages in the meat production continuum, starting with contamination of carcasses at the time of slaughter by bacteria on the skin surface of the animal, on its hooves, or even from its intestines. Poor sanitization of slaughter tools used for skinning and evisceration, poor dressing of carcasses, or other hygiene‐related issues contribute significantly to increased levels of pathogen transfer during the process. Additionally, poor conditions during chilling and storage of carcasses, which is another stage where *E. coli* can multiply if not cooled quickly or if kept in poor sanitary conditions, make it possible for this kind of contamination. Also, during secondary processing of meat products, including butchering and packaging of raw meat products such as ground beef, among others, increases cross‐contamination (Dharma et al. 2022; Diyantoro and Wardhana 2019).

The problem of antimicrobial resistance (AMR) among livestock is a major international issue that carries far‐reaching consequences not only for veterinary but also human health. Among agricultural practices, antimicrobial agents are widely used for therapy, prophylactic purposes, and growth promotion of farm animals (Islam et al. 2024). However, their irrational use results in a higher rate of formation of AMR strains (Manyi‐Loh et al. 2018). Such organisms may spread between animals and humans through various routes of exposure, including the intake of contaminated food, contamination with polluted water and soil, and usage of organic fertilizers containing livestock excreta.

Antimicrobial resistance in livestock populations presents an immediate challenge to the success of any medication used in medical treatments for humans. The presence of these resistant organisms is linked with high levels of treatment failure and long hospitalization periods. Furthermore, the transfer of resistance from animals to humans aids in spreading these microorganisms throughout the world, thus increasing the cost of managing these infections (Bava et al. 2024). From a broader perspective, the discovery of these organisms in meat and meat products often leads to the recall of these products, damaging brand names and lowering customer confidence. This causes great economic losses to the meat industry (Kurćubić et al. 2025).

Network meta‐analysis represents a well‐established statistical technique which builds on conventional meta‐analysis approaches through providing an ability to concurrently examine several treatment options using a single network of studies. In the context of AMR, NMA is especially beneficial since it allows for analyzing the effectiveness of various antimicrobials when used against particular pathogens, even in cases where direct clinical evidence is lacking in the available literature base. By integrating information from a variety of sources, such an analysis could be used to reveal resistance levels of pathogens to antimicrobials across various pharmacological classes. Although the NMA approach has already been utilized in order to evaluate antimicrobial efficacy in veterinary studies (Abdulkareem 2023; Mahmoudi et al. 2025; Sharifi et al. 2025), a significant knowledge gap has emerged since there has been no study using this method to examine resistance patterns among bacteria found in animal foods.

The study focuses on *E. coli* in beef and beef products, owing to the significance of the bacterium as a zoonotic agent involved in numerous outbreaks, especially those involving raw or uncooked beef products, and also as a principal organism that is monitored for AMR development trends in animals. Although the correct cooking of the beef reduces the biological hazards, post‐processing infection risk and the worldwide consumption of different meat types remain an issue. The AMR characteristics exhibited by *E. coli* in the beef supply chain indicate the antimicrobial pressure in modern agriculture that impacts animal welfare and human medicine alike. Thus, the research objective is to use network meta‐analysis to identify global differences in AMR among *E. coli* strains in beef products.

## 2 Methods

### 2.1 Literature Search Methodology

In conducting the NMA, the PRISMA guidelines were strictly followed to enhance scientific integrity in our study methods (Moher et al. 2009). A comprehensive search of international journals was carried out from PubMed, Springer, Wiley, Google Scholar, and Elsevier databases, covering all articles up to October 2025. No language barriers were enforced to maintain consistency and eliminate any possibility of selection bias. Specific search terms such as “antimicrobial,” “antibiotic,” “antibiogram,” “resistance,” and “susceptibility” were used in conjunction with the search terms pertaining to the host and product, namely “bovine,” “cattle,” “cow,” “buffalo,” “meat,” “beef,” and “meat products.”

### 2.2 Inclusion and Exclusion Criteria

The eligibility criteria for the inclusion of studies were based on rigorous pre‐defined guidelines. The following guidelines were followed for inclusion in the network meta‐analysis: (1) results should be expressed in terms of the percentage of resistance/susceptibility, (2) the authors had to provide the unadjusted numbers such as total isolate numbers and resistant numbers for each antimicrobial, and (3) the study design needed to allow comparisons of at least two different antimicrobials. The studies that did not include detailed quantitative information, were published solely as abstracts/reviews, had duplicated information, or were insufficiently designed experiments were not included in the analysis. All studies on the isolation of *E. coli* from various stages of the meat production process were considered in order to have a thorough analysis of the risks in public health.

### 2.3 Data Extraction and Standardization

The preliminary assessment of titles and abstracts for the selected articles was done independently to filter relevant literature, and disagreements among results obtained were resolved through discussion between P.M. and S.I.H. Following that, the selected articles were analyzed thoroughly to confirm whether or not they adhered to the set inclusion criteria. The high quality of data provided was ensured as only studies implementing standardized procedures for AST (e.g., using disc diffusion test, broth microdilution, or agar dilution) were used. Moreover, the resistance interpretation should match international guidelines of CLSI, NCCLS, or EUCAST.

In the process of systematic review and data extraction, the following information was gathered for each selected article: name of the primary author, year of publication, geographical data (country and continent where research was conducted), development status of the country, classification of *E. coli* , names and classes of used antimicrobials, number of resistant isolates, total number of isolates. In case of longitudinal research carried out for two consecutive years (such as in 2019 and 2020), the time period of investigation is marked as the first year.

For ensuring the biological relevance of *E. coli* isolates, they were classified as pathogenic in case of identification of known toxigenic virulence factors (*stx1*, *stx2*, *eae*, *ipaH*, or *hlyA*) or pathogenic *E. coli* serotypes (O157:H7, O26:H11, or O111:H8). In all other cases, isolates were classified as non‐pathogenic *E. coli* . Additional details and supporting data are provided in Data S1.

### 2.4 Statistical Analysis and Predictive Modeling

The extracted data were subjected to further analysis in order to obtain study‐specific effect size estimates, measured as odds ratios (ORs) along with their 95% credible intervals (CrI). Network meta‐analysis (NMA) was performed using R (version 4.4.1) with the aid of the gemtc package. The analysis was done according to Bayesian approach with the help of three distinct Markov Chain Monte Carlo (MCMC) chains. Burn‐in phases consisted of 5000, 15,000, and 25,000 iterations, after which came sampling phases with 10,000, 30,000, and 50,000 iterations, respectively, with the thinning interval equal to one.

Heterogeneity between studies was measured by means of the *I* ^2^ test, where *I* ^2^ above 50% indicated the presence of considerable heterogeneity, while an *I* ^2^ value below 50% denoted low heterogeneity. Based on the amount of heterogeneity present in the analysis, the fixed‐ or the random‐effects consistency model was chosen. MCMC convergence was evaluated through the application of the Gelman‐Rubin diagnostic (Gelman and Rubin 1992), and the model with the smallest Potential Scale Reduction Factor (PSRF) was preferred.

The rankings of the interventions were based on the Bayesian probabilities for ranks and presented using the Surface Under the Cumulative Ranking Curve (SUCRA). The SUCRA value is a numerical index between 0 and 1 representing the total probability that a particular intervention holds every single rank position across the whole network (Salanti et al. 2011). High SUCRA values denote a high probability of being at a better rank position, while low SUCRA values signify that a treatment is more likely to have a lower rank position. Since the outcome under investigation was antimicrobial resistance rate, a detrimental outcome, the SUCRA values were taken inversely to show the antimicrobial retention efficacy. Hence, the antimicrobials with higher SUCRA values had higher resistance rates and less efficacy while those with lower SUCRA values had lower resistance rates and high efficacy. SUCRA values present a compact description of the relative positions of all interventions in the network analysis. However, SUCRA values should always be used in connection with the effect sizes and credible intervals because having different positions does not mean significant differences between interventions. Network meta‐analysis results are expressed in terms of pooled odds ratio (OR) and 95% credible interval (CrI).

Node splitting was used to examine the consistency between indirect and direct evidence. Publication bias was examined by Egger's regression test (Egger et al. 1997). For analyzing small study effect, we did network meta‐regression with sample size (< 20 versus ≥ 20 isolates for each antimicrobial) as one of the covariates. The other covariates were *E. coli* virulence, continent of studies, and economic level of country.

Moreover, antimicrobial resistance trends were forecasted up to 2040 utilizing the Prophet forecasting method, considering the period from 2000 to 2024. It is important to mention that forecasting based on the Prophet model relies on the assumption that resistance trends will continue following the established historical path, which means that no exogenous factors, including future government policy implementation and any other changes to the existing situation, have been considered by the model in this regard.

## 3 Results

### 3.1 Study Selection Process

**FIGURE 1:**
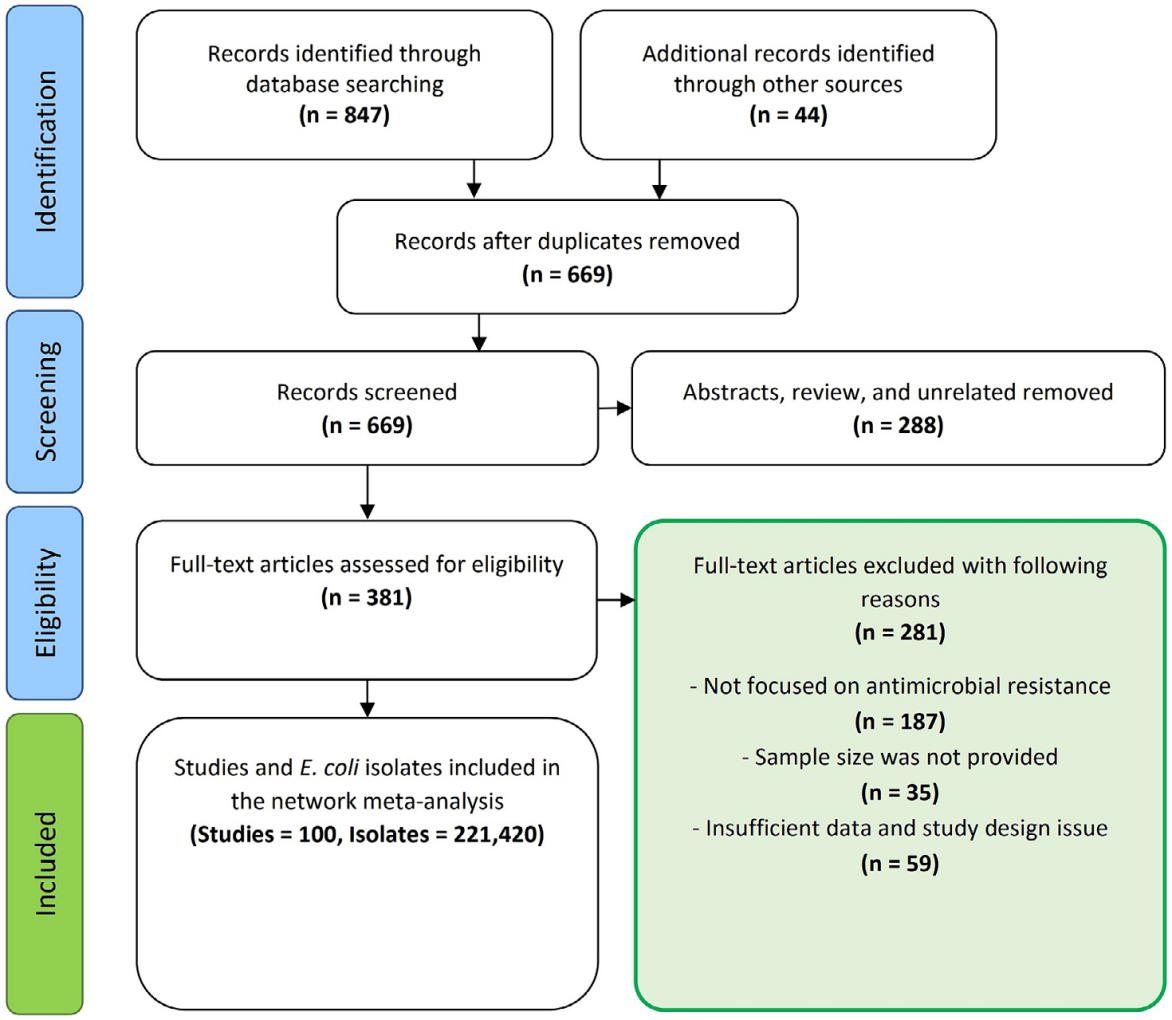
PRISMA flow diagram of study selection.

The entire procedure of searching and screening is shown in the PRISMA flow chart (Figure 1). Initially, a total number of 847 studies was extracted from the comprehensive search of databases. After eliminating duplicate studies, 288 studies were excluded owing to irrelevancy to the study topic or dissemination of non‐primary articles including reviews or abstracts. Another 281 studies were also excluded during full‐text screening based on a number of specific grounds; 187 articles failed to cover the theme of AMR, 59 studies lacked sufficient quantitative data, and 35 studies lacked necessary information about sample size. Therefore, a total number of 100 quality studies was chosen to be part of the current network meta‐analysis. Data from a large number of 221,420 isolates of *E. coli* obtained from bovine meat samples was used in the studies.

### 3.2 Characteristics of Included Studies

In total, 100 publications ranging from 1993 to 2025 met the inclusion criteria for our network meta‐analysis. At the outset, 91 different antimicrobials were identified in the literature; nevertheless, to achieve statistical stability, those investigated in less than five trials were removed. Although the aforementioned number can provide us statistically valid results, it should be noted that it could have led to excluding some of the recently developed antimicrobials like ceftazidime‐avibactam and aztreonam‐avibactam, along with regional antimicrobials, where their lack of citations is related to the geographical limitations in use and not to antimicrobial resistance.

Also, antimicrobial classes that were inherently resistant to *E. coli* (macrolides [erythromycin, azithromycin], lincosamides [lincomycin], and glycopeptides [vancomycin]) were also excluded from the final NMA model. This resulted in 39 antimicrobials as part of the core group in the NMA analysis. The tetracycline class was the most commonly studied among the 39, appearing in 86 studies; gentamicin and ampicillin were second and third, respectively, at 82 and 80 studies. The database represents a wide global distribution, with evidence from 41 countries on five different continents. Table 1 provides an extensive description of *E. coli* isolates.

**TABLE 1:** Summary of the number of *E. coli* isolates from the various categories included in this study.

Category	Number of isolates
*E. coli* type	
Pathogenic	10,006
Non‐pathogenic	211,414
Development status of country where isolates are tested	
Developed	188,329
Developing	33,091
Region	
Africa	10,750
Asia	19,221
Europe	36,560
North America	151,604
South America	3285

### 3.3 Evaluation of Heterogeneity, Inconsistency, and Publication Bias

Network meta‐analysis robustness was achieved by conducting diagnostic tests. Local inconsistency was evaluated through a node‐splitting technique, and most comparisons revealed *p* > 0.05. This showed high consistency between the direct and indirect effects and lack of any serious local inconsistencies. In addition, regression test by Egger generated *p*‐value of 0.47, implying that the results were free from any publication bias. Sensitivity tests to evaluate model robustness were performed based on excluding small studies from the dataset. The generated resistance profile was similar to the one obtained in the main analysis. Even though a few comparisons in the indirect treatment effect estimates were considered high risk of bias, the majority of the direct treatment effect estimates were moderate or high‐quality.

### 3.4 Global Network Meta‐Analysis of Antimicrobial Resistance in *E. coli*

**FIGURE 2:**
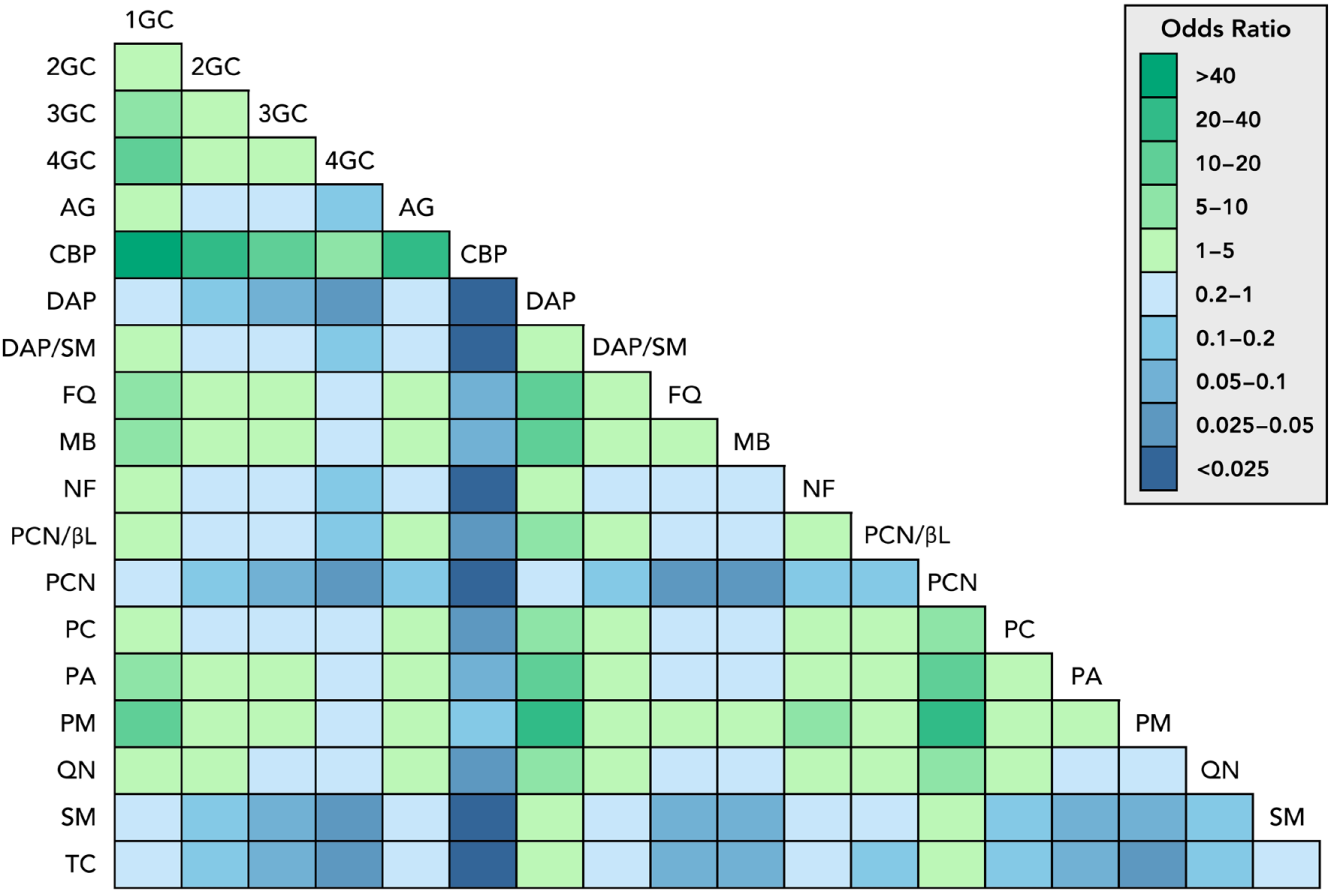
Pooled estimates of the network meta‐analysis for classes of antimicrobials: 1GC, First generation cephalosporins; 2GC, Second generation cephalosporins; 3GC, Third generation cephalosporins; 4GC, Fourth generation cephalosporins; AG, Aminoglycosides; CBP, Carbapenems; DAP, Diaminopyrimidines; DAP/SM, Diaminopyrimidines/Sulfonamides; FQ, Fluoroquinolones; MB, Monobactams; NF, Nitrofurans; PA, Phosphonic acids; PC, Phenicols; PCN, Penicillins; PCN/βL, Penicillins/β‐lactams; PM, Polymyxins; QN, Quinolones; SM, Sulfonamides; TC, Tetracyclines.

**FIGURE 3:**
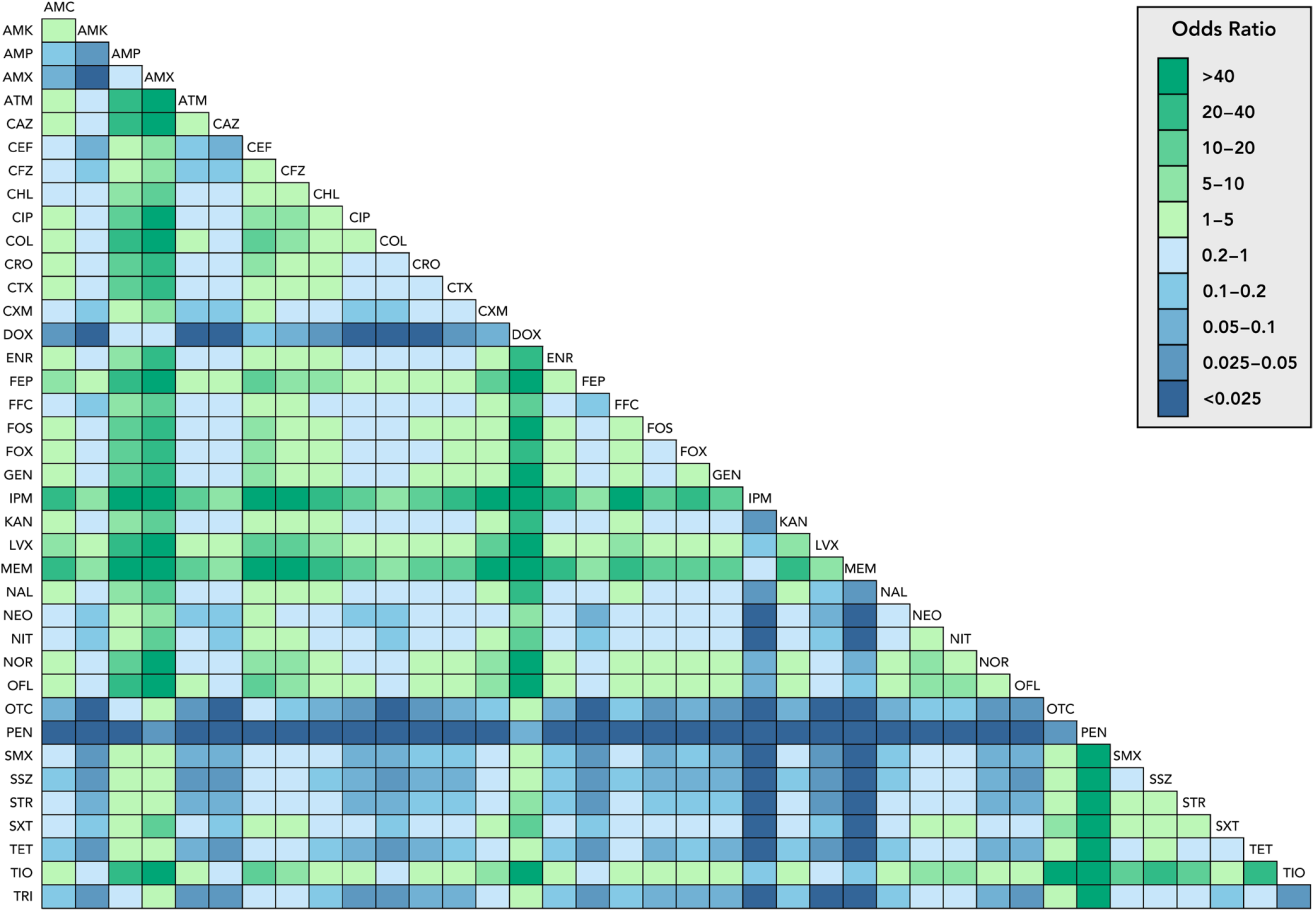
Pooled estimates of the network meta‐analysis for antimicrobials. AMC, Amoxicillin/Clavulanic Acid; AMK, Amikacin; AMP, Ampicillin; AMX, Amoxicillin; ATM, Aztreonam; CAZ, Ceftazidime; CEF, Cephalothin; CFZ, Cefazolin; CHL, Chloramphenicol; CIP, Ciprofloxacin; COL, Colistin; CRO, Ceftriaxone; CTX, Cefotaxime; CXM, Cefuroxime; DOX, Doxycycline; ENR, Enrofloxacin; FEP, Cefepime; FFC, Florfenicol; FOS, Fosfomycin; FOX, Cefoxitin; GEN, Gentamicin; IPM, Imipenem; KAN, Kanamycin; LVX, Levofloxacin; MEM, Meropenem; NAL, Nalidixic Acid; NEO, Neomycin; NIT, Nitrofurantoin; NOR, Norfloxacin; OFL, Ofloxacin; OTC, Oxytetracycline; PEN, Penicillin; SMX, Sulfamethoxazole; SSZ, Sulfisoxazole; STR, Streptomycin; SXT, Trimethoprim/Sulfamethoxazole; TET, Tetracycline; TIO, Ceftiofur; TRI, Trimethoprim.

Figures 2 and 3 and depict the OR and the corresponding 95% CrI values of pooled resistance estimates of different antimicrobial classes and specific agents. In total, a synthesis of the resistance data to 19 different antimicrobial classes and 39 agents by combining the results of direct comparisons as well as indirect estimates based on the network's statistical model was conducted.

Accordingly, this analysis has revealed that *E. coli* was significantly less resistant to imipenem than to any other antimicrobial tested, with the exception of meropenem (OR = 0.94; CrI: 0.14–6.60) and levofloxacin (OR = 0.16; CrI: 0.02–1.23). On the other hand, penicillin and doxycycline had the highest levels of resistance, being significantly more resistant than any other tested antimicrobial in the network.

On the level of antimicrobial classes, carbapenems had the lowest resistance rate, followed by polymyxins and fluoroquinolones. These antimicrobial classes demonstrated their ability to combat *E. coli* bacteria in the bovine meat production chain. On the contrary, penicillins had the highest resistance rates, with sulfonamides and tetracyclines being close in terms of resistance to the tested bacteria.

### 3.5 Rank Probabilities

**FIGURE 4:**
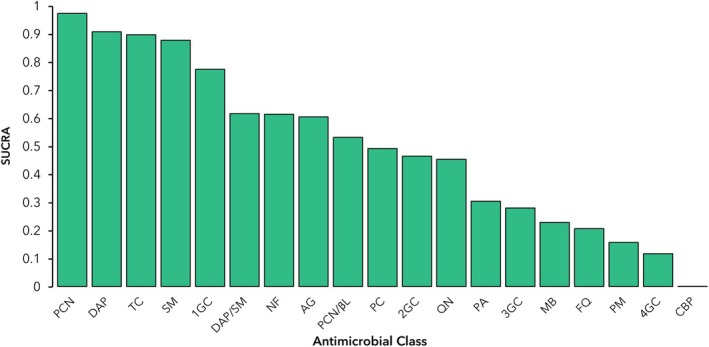
The SUCRA score of antimicrobial classes against *E. coli* . The higher SUCRA value indicates the higher resistance of *E. coli* to the corresponding class of antimicrobial. See the caption of Figure 2 for explanation of abbreviations.

**FIGURE 5:**
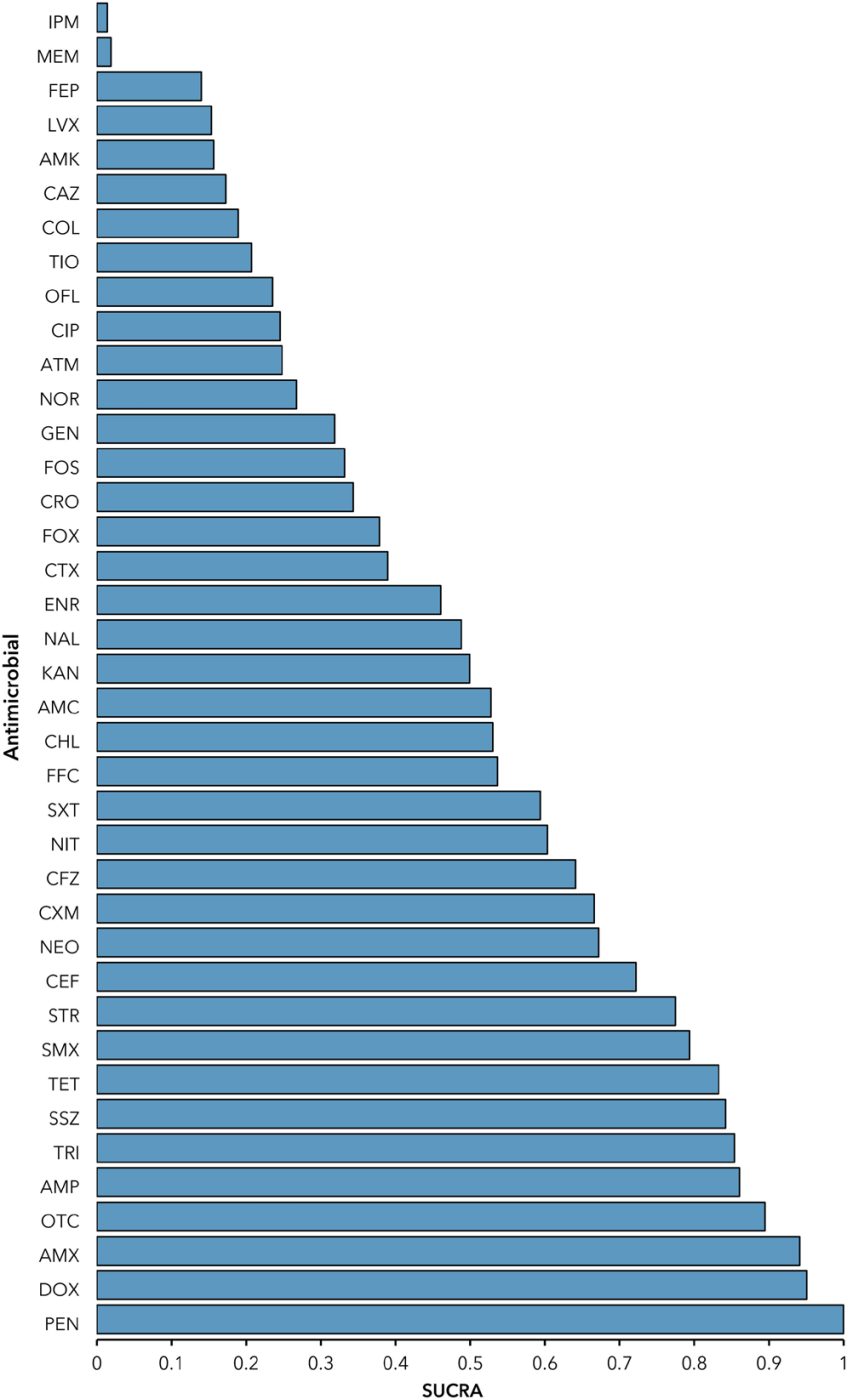
The SUCRA score of antimicrobials against *E. coli* . The higher SUCRA value indicates the higher resistance of *E. coli* to the corresponding class of antimicrobial. See the caption of Figure 3 for explanation of abbreviations.

Rank probabilities are provided in terms of the SUCRA values for classes of antimicrobials and specific antimicrobials (Figures 4 and 5). According to the results, the carbapenems and fourth‐generation cephalosporins were characterized by the lowest probability values (SUCRA = 0.0008 and SUCRA = 0.1170) and therefore were the classes with the highest effectiveness. On the other hand, the penicillins and diaminopyrimidines were among those classes with the lowest effectiveness since they were characterized by the highest values of SUCRA (0.974 and 0.908).

In turn, imipenem, meropenem, cefepime, levofloxacin, and amikacin were among the most effective individual antimicrobials since they had relatively low SUCRA (0.014, 0.019, 0.140, 0.154, and 0.157). In particular, imipenem was found to have the lowest probability value and therefore is likely to be one of the most effective agents. Finally, the highest probability values and hence the worst performance were found for penicillin (SUCRA = 0.999) and doxycycline (SUCRA = 0.951).

### 3.6 Network Meta‐Regression

Table 2 provides the values for Deviance Information Criterion (DIC) in the case of network meta‐regressions. From the results presented in Table 2, it is evident that there is a slight decrease in DIC in the case of the primary NMA analysis as compared to those of the network meta‐regression in which sample size and antimicrobial susceptibility testing standard were considered as covariates (the DIC values being 1363.31 and 1363.84 respectively). This implies that adjustment for sample size does not have much effect on the outcome of NMA. On the contrary, network meta‐regression models considering other covariates such as location of study (continent), level of development of the country, and pathogenicity of E. coli strain were seen to have better fitting to the data.

**TABLE 2:** The results of network meta‐regression for the effects of high‐risk studies on overall outcomes.

Analysis	Deviance information criterion
Primary network meta‐analysis	1363.31
Network meta‐regression 1 (sample size)	1363.77
Network meta‐regression 2 (continent where study conducted)	1362.49
Network meta‐regression 3 (development status of countries)	1360.45
Network meta‐regression 4 (pathogenicity of *E. coli* )	1361.78
Network meta‐regression 4 (antimicrobial susceptibility testing standard; CLSI vs. EUCAST)	1363.84
Multifactor network meta‐regression	1359.88

*Note:* Model with lower Deviance Information Criterion fits data better.

### 3.7 Resistance of Pathogenic and Non‐Pathogenic *E. coli*

**FIGURE 6:**
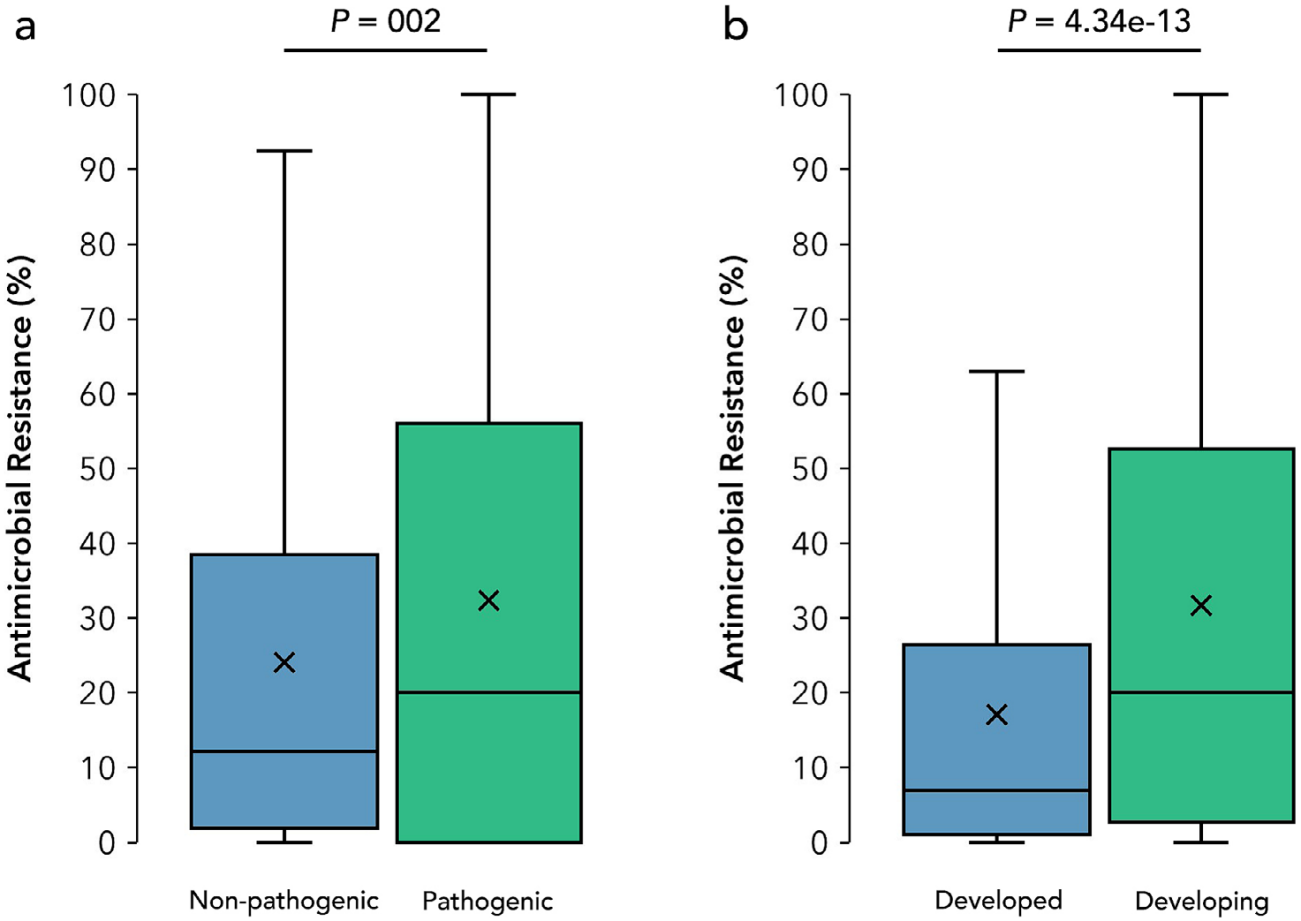
The boxplot of resistance of non‐pathogenic and pathogenic *E. coli* to antimicrobials (a), and resistance of *E. coli* to antimicrobials in developed and developing countries (b).

Figure 6a displays a boxplot comparing the antimicrobial resistance of pathogenic and non‐pathogenic *E. coli* strains. Pathogenic *E. coli* exhibited significantly higher resistance (32.35%) compared to non‐pathogenic strains (24.09%) (*p* = 0.002).

### 3.8 *E. coli* Resistance to Antimicrobials

In Table 3, there is a worldwide evaluation of antimicrobial resistance among strains of *E. coli* found in meat and meat products, where the highest and lowest percentages of resistance to particular antimicrobial agents are listed. Figure 6b shows a boxplot for the comparison of resistance among *E. coli* strains from developed and developing countries. The highest mean resistance was found in Africa (33.81%), then Asia (29.17%) and South America (22.22%). North America had the lowest resistance (18.26%). In both Africa and South America, imipenem was the antimicrobial least resistant to *E. coli* . Besides, strains from developed countries showed significantly lower levels of resistance (17.03%), compared to developing countries (31.73%) (*p* = 4.34 × 10^−13^).

**TABLE 3:** Global antimicrobial resistance of *E. coli* isolated from meat and related products and antimicrobials with the highest and lowest resistance rates against *E. coli* in each region.

Region	Average resistance (%)^a^	Highest resistance rate		Lowest resistance rate	
		Antimicrobial	Resistance (%)	Antimicrobial	Resistance (%)
Africa	33.81	Ampicillin	58.75	Imipenem	0.78
		Cefuroxime	58.03	Cefoxitin	2.77
		Amoxicillin	44.83	Amikacin	9.81
Asia	29.17	Penicillin	100.00	Fosfomycin	2.22
		Doxycycline	77.99	Imipenem	3.77
		Oxytetracycline	64.16	Colistin	4.90
Europe	20.10	Tetracycline	45.52	Cefotaxime	0.43
		Sulfamethoxazole	43.64	Ceftazidime	1.67
		Sulfisoxazole	36.00	Imipenem	3.10
North America	18.26	Nitrofurantoin	48.08	Ciprofloxacin	1.62
		Cefotaxime	43.48	Ceftiofur	3.47
		Cefalothin	37.43	Cefoxitin	4.19
South America	22.22	Cefalothin	59.88	Imipenem	0.00
		Cefotaxime	58.00	Chloramphenicol	3.85
		Nalidixic Acid	48.75	Amikacin	7.96

^a^ Average resistance refers to the average proportion of *E. coli* isolates resistant to at least one tested antibiotic within a given dataset.

### 3.9 Global Trends in Antimicrobial Resistance of *E. coli*

**FIGURE 7:**
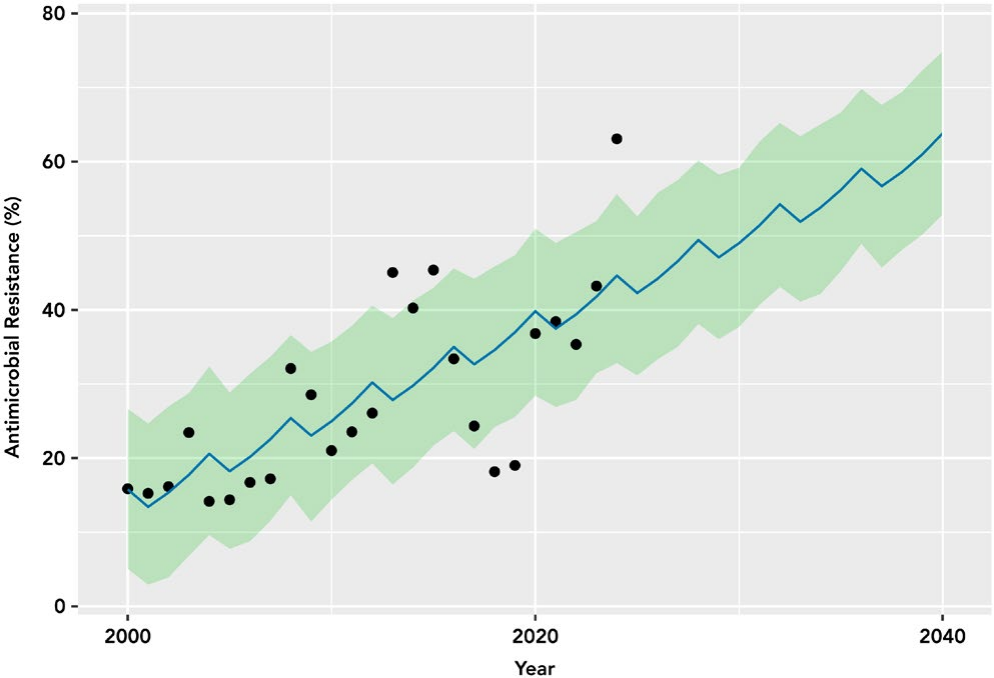
The forecasted resistance of *E. coli* isolated from bovine meat and its products until 2040.

Figure 7 presents the projected antimicrobial resistance of *E. coli* isolated from bovine meat and its products. The results indicate that the global mean resistance of *E. coli* is expected to reach 54.25% by 2030, 56.70% by 2035, and 63.44% by 2040.

### 3.10 Antimicrobial Classes Resistance Correlation

**FIGURE 8:**
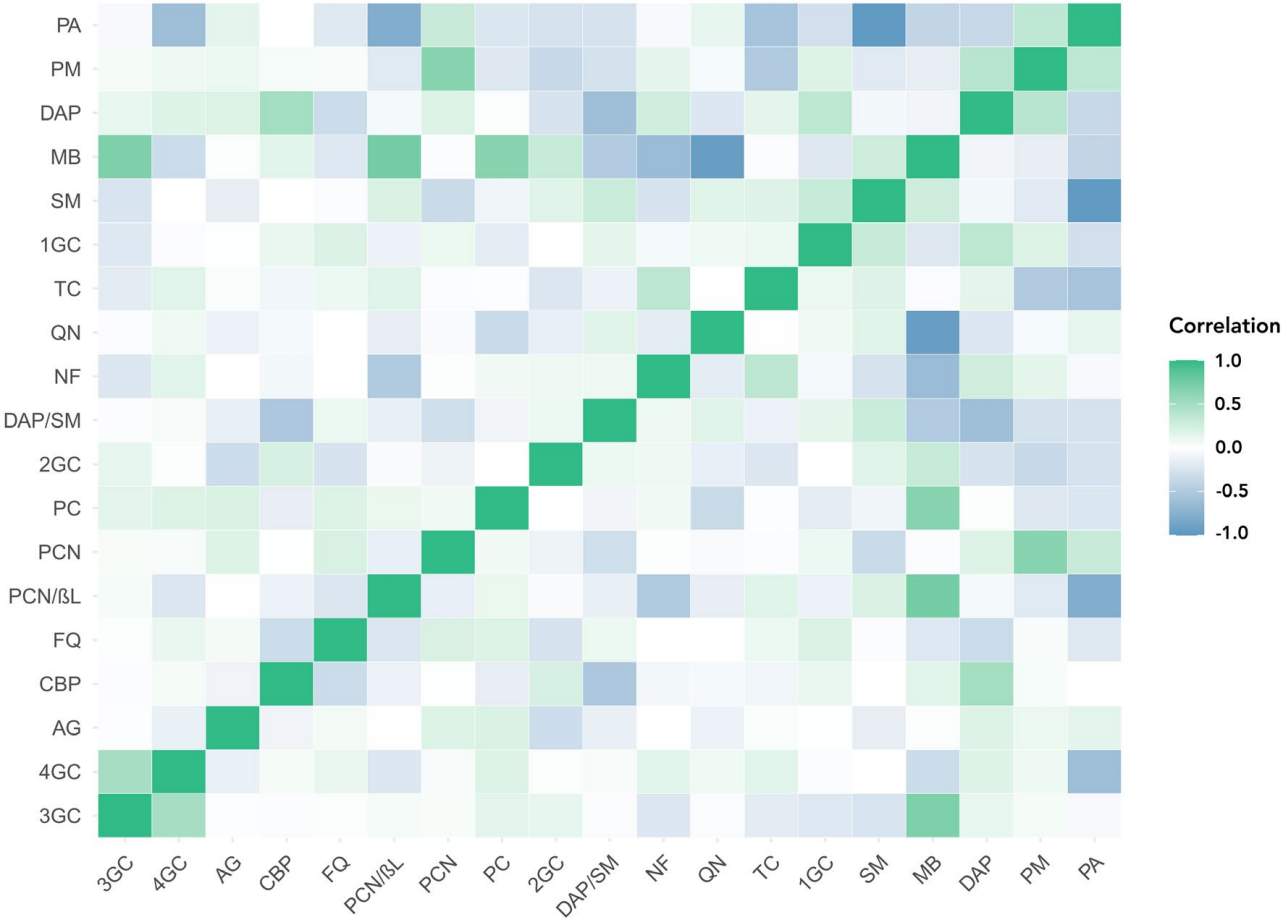
Spearman correlation heatmap of antimicrobial classes resistance patterns in *E. coli* isolated from meat and its products. See the caption of Figure 2 for explanation of abbreviations.

The Spearman correlation coefficients among resistance patterns for various types of antimicrobials are shown in Figure 8. A strong positive correlation was observed between third‐generation monobactams and cephalosporins (*r* = 0.698), as well as between monobactams and penicillins/β‐lactams (*r* = 0.742). Conversely, strong negative correlations were identified between phosphonic acids and penicillins/β‐lactams (*r* = −0.789), phosphonic acids and sulfonamides (*r* = −0.975), and between monobactams and quinolones (*r* = −0.938).

## 4 Discussion

The results of the present network meta‐analysis suggest that there is a relatively high prevalence of antimicrobial resistance to *E. coli* isolated from beef and associated products. Based on the findings, *E. coli* shows considerable insensitivity to various essential therapeutics including ampicillin, amoxicillin, penicillin, oxytetracycline, and doxycycline. As far as the penicillin subclass of antimicrobials is concerned, resistance rates are relatively high. Penicillin demonstrated the highest SUCRA score of 0.999 while the scores of other agents included in this group are also relatively high: 0.942 (amoxicillin) and 0.861 (ampicillin). Such resistance rates raise concerns regarding the potential ineffectiveness of these medications in cases when infections caused by *E. coli* originate in beef products. However, resistance has also been observed in the cases of tetracycline and diaminopyrimidine antimicrobials. The mentioned results indicate the ever‐growing problem of antimicrobial resistance that is of great importance not only from the perspective of human health but also veterinary medicine.

In contrast, the current study revealed low resistance levels among *E. coli* strains against carbapenems, namely imipenem and meropenem, as well as against fourth‐generation cephalosporins (cefepeime) and polymyxins (colistin). However, it is worth mentioning that the low prevalence rates of carbapenem resistance most probably are connected to their classification as critically important antimicrobials in human therapy and subsequent prohibition of their usage on livestock in the majority of countries worldwide. Lack of access to such antimicrobials constitutes one of the main factors that prevent bacteria from developing resistance. While misuse might happen in countries with weak legislation, the use of these antimicrobials in animals is generally prohibited. Thus, the results of the research emphasize the need for increasing antimicrobial regulation standards across the globe and adopting responsible policies towards their use.

This research does not endeavor to generate recommendations regarding the use of antimicrobials in the treatment of animals in veterinary settings or humans in the healthcare setting. Instead, the current study aims to shed light on the international prevalence of resistance to antimicrobials exhibited by *E. coli* isolated from bovine meat. The findings reveal the importance of resistant *E. coli* in terms of public health concerns, as well as dangerous methods of infection through consumption of contaminated foods, posing hazards to human health. The results of the study emphasize the critical need for continuous surveillance activities as well as proper implementation of stewardship programs. This study also encourages adherence to laws and regulations on a national and international level, which are vital to curb resistant pathogens and maintain priority antimicrobials in the global medical armamentarium.

The increase in resistance frequency against penicillins and diaminopyrimidines seen in this study is probably due to the widespread use of these antimicrobials in human healthcare and veterinary medicine. The use of penicillins like amoxicillin and ampicillin is one of the most common treatments used in livestock care. This significant use creates selection pressure, favoring the development of resistance among certain bacterial strains (Llor and Bjerrum 2014). Likewise, the extensive and long‐term use of diaminopyrimidines in animal agriculture for several decades allowed for the spread of resistance genes. These transfers have largely been facilitated through the process of horizontal gene transfer. Mobile genetic elements like transposons and plasmids play critical roles in transferring resistant genes between bacteria (Guernier‐Cambert et al. 2021).

From the analysis, there is substantial evidence of a significant difference in the antimicrobial resistance patterns of both pathogenic and commensal *E. coli* strains. These differences arise owing to various interconnected ecological, genetic, and molecular processes. Pathogenic strains, as the causative pathogens for infections, undergo more selective pressure because they encounter antimicrobials frequently used in medical treatments in humans and animals. Consequently, these pathogens acquire more antimicrobial resistance genes, which disseminate further among the pathogenic strains. It is interesting that pathogenic *E. coli* strains can acquire resistance determinants more easily compared to other bacterial strains. Pathogenicity islands (PAIs), which are genomic regions encoding virulence genes, co‐localize with the genes that encode antimicrobial resistance and contain structural properties similar to those found on mobile elements. Thus, PAIs are mobilizable together with the resistance genes (Naidoo and Zishiri 2025; Zakaria et al. 2022).

Additionally, several pathogen strains bear conjugative plasmids and ICEs that, in turn, harbor both virulent and antimicrobial resistant genes, thus permitting the conjugation of the entire resistance/virulence gene cluster as a genetic unit under common selective pressure (Rozwandowicz et al. 2018). The fact indicates that the action of antimicrobial agents can select for both resistance and virulence among the pathogenic strain population. OMVs generated by pathogenic *E. coli* are another yet largely neglected mode of resistance spread because OMVs can transfer resistance plasmids and chromosomes to other cells even without cell–cell contact (Chatterjee et al. 2017). On the contrary, nonpathogenic strains rarely face exposure to antimicrobial substances, and when exposed, the latter appear in low amounts, which, as a result, leads to a low selective pressure and a low level of resistance among the strain population (Harada and Asai 2010). It is evident from the foregoing that there exists a significant difference between pathogenic and nonpathogenic strains' antimicrobial exposure profiles. The former is characterized by high selective pressure, while the latter displays low selective pressure, thus pointing to the major effect of clinical usage patterns, along with the genetic flexibility of pathogenic strains, on resistance evolution.

The current analysis illustrates the geographical variation in AMR profiles in terms of isolates of *E. coli* taken from meat products derived from cattle slaughtered in the developing and developed world. From the quantitatively determined Figures, it is clear that the frequency of AMR in developing nations (31.73%) is significantly higher than the frequency of AMR in developed countries (17.03%). It is important to note that the highest average continent frequency is observed in Africa (33.81%). The reasons for this discrepancy include several factors that are interconnected with one another. First, the increased use of antimicrobials for growth promotion and prophylactic purposes among the farming communities of developing countries contributes greatly to this phenomenon (Ayukekbong et al. 2017). Additionally, the weak regulation concerning the implementation of policies related to the use of veterinary pharmaceuticals in developing nations also accounts for the problem. This factor, along with poor IPC practices in both medical institutions and farms, results in the misuse of antimicrobial medications in question (Pyatt et al. 2025). Poor sanitation infrastructure and inadequate hygiene practices during meat processing and handling also contribute to the emergence of AMR as a result of the easy spread of the bacteria (IssaZacharia et al. 2025).

In addition to selective pressure, the propagation of multidrug resistance (MDR) among *E. coli* populations in bovines is heavily dependent upon co‐selection processes involving mobile genetic elements (MGEs). Plasmids, specifically Incompatibility Group IncF, IncI, and IncN types, have been highlighted as major factors in the co‐selection of various resistance markers in the case of *E. coli* in food‐producing animals, facilitating the transfer of resistance genes for diverse antimicrobial classes through a single process of conjugation (Rozwandowicz et al. 2018). This factor contributes to the significant positive correlation between various subclasses of β‐lactams identified in the current study (Figure 8) because the co‐presence of such resistance genes on similar plasmids should result in comparable prevalence trends across different populations. Another important MGE for the selection of resistance genes is integrases, particularly class 1 integrons, which show high prevalence in agroecosystems. Integrases provide another platform for resistance cassettes that can combine genes of resistance against aminoglycosides, sulfonamides, and trimethoprim into one operon (Partridge et al. 2018).

The presence of co‐selection pressures for resistance genes due to association with integrons and plasmids within livestock‐derived strains is another co‐selection pressure factor operating outside the scope of antimicrobial utilization and contributing to maintenance of resistance even when there is no antimicrobial treatment at all (Pal et al. 2015). In addition, transposons, insertion sequences, and genomic islands increase the flexibility of the genome of bovine *E. coli* by facilitating the transfer of resistance genes between plasmids and chromosomal DNA, fixation of resistance genes in the core genome, and decreasing the selection costs of maintaining them. In summary, the discussed mechanisms indicate that resistance management in the context of beef production should involve more than limiting antimicrobial use since it is not possible to control the co‐selection pressures created by heavy metals and biocides.

Spearman correlation test of *E. coli* resistance to the different antimicrobials indicated the existence of many significant relationships. There was a significant positive correlation between third‐generation monobactams and cephalosporins. It is suggested that these antimicrobials have a similar mechanism of resistance development due to the fact that both are β‐lactam antimicrobial agents. Another significant positive correlation was found between monobactams and penicillins/β‐lactams, which indicates their close ecological relationship in terms of resistance to the antimicrobials. Many significant negative correlations were found. Resistance to phosphonic acids had a significant negative correlation with resistance to both penicillins/β‐lactams and sulfonamides. It might be explained by different patterns of resistance development and different use frequency. Monobactams were also negatively correlated with resistance to quinolones, which indicates that monobactams and quinolones have different resistance profiles.

The strongest associations are noted to have clustering in β‐lactam‐related clusters but are seen to differ from those related to resistance for β‐lactams and non‐β‐lactam antimicrobials. This highlights that there is a naturally heterogeneous pattern of *E. coli* resistance in different pharmacological classes of antimicrobials.

One major concern regarding NMA is the small sample sizes in some of the studies included. In order to determine whether there is any impact of sample size on the overall outcome, a network meta‐regression was conducted to examine the presence of bias based on study samples. Based on the analyses, it can be noted that the DIC of the primary analysis is significantly lower than that for sample size‐adjusted regression. Therefore, it can be concluded that there is no significant bias due to the effect of different sample sizes.

A related limitation relates to the restriction to antimicrobial compounds studied in less than five studies. Although this restriction is intended to ensure the validity of estimates within the network, it necessarily involves a certain degree of coverage bias. The particular limitation includes the underrepresentation of the patterns of antimicrobial resistance in novel β‐lactam/β‐lactamase inhibitor pairs (such as ceftazidime‐avibactam and ceftolozane‐tazobactam) and last‐line drugs (such as tigecycline). Although these antimicrobials are highly clinically significant, they have been poorly investigated concerning the development of antimicrobial resistance in food‐animal associated strains of *E. coli* . Moreover, region‐specific antimicrobials, including enrofloxacin pairings and local versions of β‐lactams, may not be adequately captured. Future network meta‐analyses should investigate the potential for these patterns of resistance emergence as the literature develops.

Prophet is useful in forecasting antimicrobial resistance because it can accommodate missing data, detect shifts in trends, and capture seasonal effects. However, the projections produced by this model should be carefully interpreted since it assumes that the resistance trends witnessed between 2000 and 2024 will remain unaltered. Such a forecast is not guaranteed since effective implementation of antimicrobial stewardship programs, WHO efforts to curb AMR, and additional regulations may render it inaccurate. This model also does not take into consideration any new resistance mechanisms or new types of resistance genes that can emerge in the coming years. The selection of countries and periods covered by different studies is highly skewed; thus, it is likely to favor forecasts for North America and Europe, where more research has been conducted. As such, one should treat the global AMR rate projected at 63.44% in 2040 as the baseline scenario, rather than as the most probable scenario.

Geographical sampling bias could arise from the disparity among the distribution of studies conducted on the continents, which is reflected in the greater proportion of isolates from North America (151,604 out of 221,420; see Table 1), as well as in the relative lower number of isolates from South America and Africa.

One of the main challenges in network meta‐analysis (NMA) is the inconsistency between direct and indirect evidence, which may affect the accuracy in estimating and interpreting comparative effects of treatments (van Valkenhoef et al. 2016). In order to solve this problem, this study performed a thorough investigation of inconsistency via a node‐splitting model. The outcome of the study showed that the direct evidence and most of the indirect evidence had agreement, corresponding to the overall consistency model. Although some differences existed among a small number of indirect evidence, no inconsistency was observed because the *p*‐values did not indicate any significance (*p* > 0.05).

When considered in totality, the findings described above identify the present prevalence levels of antimicrobial resistance among *E. coli* strains associated with bovine meat production and project their future trend under existing circumstances. Future research should therefore focus on longitudinal studies using standardized methodology within regions that are currently underserved, including analysis of genetic factors that contribute to resistance and development of mathematical models to simulate the impact of appropriate stewardship measures on the evolution of resistance rates. This is particularly important for converting the general trends observed here to concrete policy solutions at the regional level.

## 5 Conclusions

This network meta‐analysis provides an overall picture of the growing problem of antimicrobial resistance worldwide in *E. coli* isolates collected from bovine meat products. The analysis demonstrates significant differences in levels of resistance, depending on both pharmacologic group of the antimicrobial agent used and socio‐economic status of the area. High resistance towards penicillin and tetracycline correlates with high utilization of antimicrobial agents among cattle rearing populations globally, although regional variations exist with regard to the frequency of use, regulations, as well as reasons for administering these agents (treatment vs. prophylactic vs. growth promoting), which have to be carefully considered when making any conclusions about the use of antimicrobial agents in practice. Conversely, low resistance towards carbapenems suggests that restrictions on their utilization can be effective in maintaining their availability as “critically important” drugs for medical application. Significant difference between pathogenic and commensal bacteria, as well as a two‐fold increase in resistance in developing countries, indicates that regionally focused actions and appropriate resources are needed. Considering that the predicted prevalence of resistance will be 63.44% in 2040, such results create a compelling argument for the implementation of One Health‐based approaches to addressing the issues of antimicrobial resistance.

## Author Contributions

**Peyman Mahmoudi:** conceptualization, investigation, methodology, formal analysis, visualization, writing – original draft. **Amir Rashidi:** data curation, writing – review and editing, validation. **Jalal Rostamzadeh:** validation, data curation, writing – review and editing. **Mohammad Razmkabir:** data curation, validation, writing – review and editing. **Skala Idrees Hama Faraj:** conceptualization, writing – review and editing, data curation, formal analysis. **Yaser Khorram Del:** investigation, data curation, writing – original draft, validation.

## Funding

This authors have nothing to report.

## Disclosure

All authors have read and approved the final version of the manuscript. Corresponding Author had full access to all of the data in this study and takes complete responsibility for the integrity of the data and the accuracy of the data analysis.

## Ethics Statement

As this study is a network meta‐analysis of previously published research, it did not require ethical approval or informed consent.

## Consent

The authors have approved the manuscript for submission.

## Conflicts of Interest

The authors declare no conflicts of interest.

## Supporting information


**Data S1:** Characteristics of the studies included in the network meta‐analysis and the data extracted from each study.

## Data Availability

The authors confirm that the data supporting the findings of this study are available within the Data S1.

## References

[fsn372136-bib-0001] Abdulkareem, Z. A. 2023. “Network Meta‐Analysis of the Therapeutic Effects of Various Antibiotics on Footrot in Sheep and Cattle.” Research in Veterinary Science 160: 55–61. 10.1016/j.rvsc.2023.05.011. 37270939

[fsn372136-bib-0002] Ayukekbong, J. A. , M. Ntemgwa , and A. N. Atabe . 2017. “The Threat of Antimicrobial Resistance in Developing Countries: Causes and Control Strategies.” Antimicrobial Resistance & Infection Control 6, no. 1: 47. 10.1186/s13756-017-0208-x. 28515903 PMC5433038

[fsn372136-bib-0003] Bava, R. , F. Castagna , C. Lupia , et al. 2024. “Antimicrobial Resistance in Livestock: A Serious Threat to Public Health.” Antibiotics 13, no. 6: 551. 10.3390/antibiotics13060551. 38927217 PMC11200672

[fsn372136-bib-0004] Chatterjee, S. , A. Mondal , S. Mitra , and S. Basu . 2017. “ *Acinetobacter baumannii* Transfers the blaNDM‐1 Gene via Outer Membrane Vesicles.” Journal of Antimicrobial Chemotherapy 72, no. 8: 2201–2207. 10.1093/jac/dkx131. 28505330

[fsn372136-bib-0005] Dharma, E. , H. Haryono , A. Salman , P. Rahayu , and W. S. Nugroho . 2022. “Impact of Hygiene and Sanitation in Ruminant Slaughterhouses on the Bacterial Contamination of Meat in Central Java Province, Indonesia.” Vet. World 15, no. 9: 2348–2356. 10.14202/vetworld.2022.2348-2356. 36341075 PMC9631358

[fsn372136-bib-0006] Diyantoro, D. , and D. K. Wardhana . 2019. “Risk Factors for Bacterial Contamination of Bovine Meat During Slaughter in Ten Indonesian Abattoirs.” Veterinary Medicine International 2019: 1–6. 10.1155/2019/2707064. PMC6885763 31827760

[fsn372136-bib-0007] Egger, M. , G. D. Smith , M. Schneider , and C. Minder . 1997. “Bias in Meta‐Analysis Detected by a Simple, Graphical Test.” BMJ 315, no. 7109: 629–634. 10.1136/bmj.315.7109.629. 9310563 PMC2127453

[fsn372136-bib-0031] Food and Agriculture Organization of the United Nations . 2023. “FAOSTAT Food and Diet” [Data Set]. https://www.fao.org/faostat.

[fsn372136-bib-0008] Gambushe, S. M. , O. T. Zishiri , and M. E. el Zowalaty . 2022. “Review of *Escherichia coli* O157:H7 Prevalence, Pathogenicity, Heavy Metal and Antimicrobial Resistance, African Perspective.” Infection and Drug Resistance 15: 4645–4673. 10.2147/IDR.S365269. 36039321 PMC9420067

[fsn372136-bib-0009] Gelman, A. , and D. B. Rubin . 1992. “Inference From Iterative Simulation Using Multiple Sequences.” Statistical Science 7, no. 4: 457–472. 10.1214/ss/1177011136.

[fsn372136-bib-0010] Geurtsen, J. , M. de Been , E. Weerdenburg , A. Zomer , A. McNally , and J. Poolman . 2022. “Genomics and Pathotypes of the Many Faces of *Escherichia coli* .” FEMS Microbiology Reviews 46, no. 6: fuac031. 10.1093/femsre/fuac031. 35749579 PMC9629502

[fsn372136-bib-0011] Govindarajan, D. K. , B. M. Eskeziyaw , K. Kandaswamy , and D. Y. Mengistu . 2024. “Diagnosis of Extraintestinal Pathogenic *Escherichia coli* Pathogenesis in Urinary Tract Infection.” Current Research in Microbial Sciences 7: 100296. 10.1016/j.crmicr.2024.100296. 39553200 PMC11565050

[fsn372136-bib-0012] Guernier‐Cambert, V. , J. Trachsel , J. Maki , et al. 2021. “Natural Horizontal Gene Transfer of Antimicrobial Resistance Genes in Campylobacter Spp. From Turkeys and Swine.” Frontiers in Microbiology 12: 732969. 10.3389/fmicb.2021.732969. 34646252 PMC8504540

[fsn372136-bib-0013] Harada, K. , and T. Asai . 2010. “Role of Antimicrobial Selective Pressure and Secondary Factors on Antimicrobial Resistance Prevalence in *Escherichia coli* From Food‐Producing Animals in Japan.” Journal of Biomedicine and Biotechnology 2010: 1–12. 10.1155/2010/180682. PMC2879543 20589071

[fsn372136-bib-0014] Islam, M. , P. Bose , M. Rahman , et al. 2024. “A Review of Antimicrobial Usage Practice in Livestock and Poultry Production and Its Consequences on Human and Animal Health.” Journal of Advanced Veterinary and Animal Research 11, no. 3: 675–685. 10.5455/javar.2024.k817. 39605760 PMC11590583

[fsn372136-bib-0015] IssaZacharia, A. , M. Ghosse , J. Matondo , H. Muhimbula , and H. Saleh . 2025. “Food Safety Challenges Related to Meat Fish and Poultry Handling and Processing in Developing Countries.” Food Safety and Packaging 1, no. 2: 57–75. 10.30466/fsp.2025.56349.1010.

[fsn372136-bib-0016] Kurćubić, V. S. , M. D. Munjić , M. P. Dmitrić , S. Živković , S. B. Stajić , and I. Tomasevic . 2025. “Bacterial Antimicrobial Resistance in Meat Products—Current Concepts.” Food 14, no. 16: 2792. 10.3390/foods14162792. PMC12386145 40870707

[fsn372136-bib-0017] Llor, C. , and L. Bjerrum . 2014. “Antimicrobial Resistance: Risk Associated With Antibiotic Overuse and Initiatives to Reduce the Problem.” Therapeutic Advances in Drug Safety 5, no. 6: 229–241. 10.1177/2042098614554919. 25436105 PMC4232501

[fsn372136-bib-0018] Mahmoudi, P. , A. Rashidi , and S. Idrees Hama Faraj . 2025. “Network Meta‐Analysis of the Prevalence and Antimicrobial Resistance of *Escherichia coli* Isolated From Bovine Milk and Dairy Products: A Global Perspective.” Journal of Dairy Science 108, no. 9: 9330–9344. 10.3168/jds.2024-26197. 40639657

[fsn372136-bib-0019] Manyi‐Loh, C. , S. Mamphweli , E. Meyer , and A. Okoh . 2018. “Antibiotic Use in Agriculture and Its Consequential Resistance in Environmental Sources: Potential Public Health Implications.” Molecules 23, no. 4: 795. 10.3390/molecules23040795. 29601469 PMC6017557

[fsn372136-bib-0032] Moher, D. , A. Liberati , J. Tetzlaff , D. G. Altman , and The PRISMA Group . 2009. “Preferred Reporting Items for Systematic Reviews and Meta‐Analyses: The PRISMA Statement.” PLoS Medicine 6, no. 7: e1000097. 10.1371/journal.pmed.1000097. 19621072 PMC2707599

[fsn372136-bib-0020] Naidoo, N. , and O. T. Zishiri . 2025. “Presence, Pathogenicity, Antibiotic Resistance, and Virulence Factors of *Escherichia coli* : A Review.” Bacteria 4, no. 1: 16. 10.3390/bacteria4010016.

[fsn372136-bib-0021] Pal, C. , J. Bengtsson‐Palme , E. Kristiansson , and D. G. J. Larsson . 2015. “Co‐Occurrence of Resistance Genes to Antibiotics, Biocides and Metals Reveals Novel Insights Into Their co‐Selection Potential.” BMC Genomics 16: 964. 10.1186/s12864-015-2153-5. 26576951 PMC4650350

[fsn372136-bib-0022] Partridge, S. R. , S. M. Kwong , N. Firth , and S. O. Jensen . 2018. “Mobile Genetic Elements Associated With Antimicrobial Resistance.” Clinical Microbiology Reviews 31, no. 4: e00088‐17. 10.1128/CMR.00088-17. 30068738 PMC6148190

[fsn372136-bib-0023] Pyatt, A. Z. , S. Eckford , N. Joseph , S. P. Borriello , and O. Oyati . 2025. “Veterinary Medicinal Product Regulation in Sub‐Saharan Africa: Identifying Barriers and Opportunities for Enhancing VMP Regulatory Systems.” Frontiers in Veterinary Science 12: 1532098. 10.3389/fvets.2025.1532098. 40417371 PMC12100746

[fsn372136-bib-0024] Rozwandowicz, M. , M. S. M. Brouwer , J. Fischer , et al. 2018. “Plasmids Carrying Antimicrobial Resistance Genes in Enterobacteriaceae.” Journal of Antimicrobial Chemotherapy 73, no. 5: 1121–1137. 10.1093/jac/dkx488. 29370371

[fsn372136-bib-0025] Salanti, G. , A. E. Ades , and J. P. A. Ioannidis . 2011. “Graphical Methods and Numerical Summaries for Presenting Results From Multiple‐Treatment Meta‐Analysis: An Overview and Tutorial.” Journal of Clinical Epidemiology 64, no. 2: 163–171. 10.1016/j.jclinepi.2010.03.016. 20688472

[fsn372136-bib-0026] Sharifi, A. , P. Mahmoudi , K. Sobhani , and M. Ashengroph . 2025. “The Prevalence and Comparative Analysis of Adhesion and Biofilm‐Related Genes in *Staphylococcus aureus* Isolates: A Network Meta‐Analysis.” Microbiology and Immunology 69, no. 2: 104–113. 10.1111/1348-0421.13189. 39639432

[fsn372136-bib-0027] van Valkenhoef, G. , S. Dias , A. E. Ades , and N. J. Welton . 2016. “Automated Generation of Node‐Splitting Models for Assessment of Inconsistency in Network Meta‐Analysis.” Research Synthesis Methods 7, no. 1: 80–93. 10.1002/jrsm.1167. 26461181 PMC5057346

[fsn372136-bib-0030] World Health Organization . 2024. “Diarrhoeal Disease.” https://www.who.int/news‐room/fact‐sheets/detail/diarrhoeal‐disease.

[fsn372136-bib-0028] Yu, D. , G. Banting , and N. F. Neumann . 2021. “A Review of the Taxonomy, Genetics, and Biology of the Genus *Escherichia* and the Type Species *Escherichia coli* .” Canadian Journal of Microbiology 67, no. 8: 553–571. 10.1139/cjm-2020-0508. 33789061

[fsn372136-bib-0029] Zakaria, A. S. , E. A. Edward , and N. M. Mohamed . 2022. “Pathogenicity Islands in Uropathogenic *Escherichia coli* Clinical Isolate of the Globally Disseminated O25:H4‐ST131 Pandemic Clonal Lineage: First Report From Egypt.” Antibiotics 11, no. 11: 1620. 10.3390/antibiotics11111620. 36421264 PMC9686529

